# Absence of the Appendix Vermiformis

**Published:** 1903-10

**Authors:** 


### ABSENCE OF THE APPENDIX VERMIFORMIS.

MICHAUX, (*Virginia Medical Semi-monthly,* Sept. 12, 1902), reports two cases in which the diagnosis of appendicitis had been made where operation showed no appendix to exist. In one a perinephritic abscess was found under the colon and behind the peritoneum. In the second case the right ovary was cystic.

